# How Optimism Bias and Safety Climate Influence the Risk-Taking Behavior of Construction Workers

**DOI:** 10.3390/ijerph19031243

**Published:** 2022-01-22

**Authors:** Siu Shing Man, Ruifeng Yu, Tingru Zhang, Alan Hoi Shou Chan

**Affiliations:** 1 Shenzhen Research Institute, City University of Hong Kong, Shenzhen 518060, China; ssman6-c@my.cityu.edu.hk (S.S.M.); meachan@cityu.edu.hk (A.H.S.C.); 2 Department of Advanced Design and Systems Engineering, City University of Hong Kong, Hong Kong 518057, China; 3 Department of Industrial Engineering, Tsinghua University, Beijing 100084, China; yurf@tsinghua.edu.cn; 4 Institute of Human Factors and Ergonomics, Shenzhen University, Shenzhen 518060, China

**Keywords:** construction safety, construction workers, optimism bias, risk-taking behavior, safety climate

## Abstract

Risk taking among construction workers is a critical topic in construction safety research. The aim of this study was to empirically investigate how optimism bias and safety climate influence construction worker risk-taking behavior. A survey with a designed questionnaire was conducted to collect data from construction workers. A total of 183 construction workers participated in this study and completed the designed questionnaire. The collected data were subjected to statistical analysis by using structural equation modeling. Results show that optimism bias related to work risks positively influences construction worker risk-taking behavior, whereas safety climate and optimism bias related to hazard perception skills negatively affect the risk-taking behavior. These findings can enrich the literature on construction worker risk-taking behavior from the perspective of optimism bias and safety climate. Practical implications are provided for discouraging construction workers from taking risks at work.

## 1. Introduction

Undoubtedly, the construction industry is a dangerous industry because of the high risk of injuries and fatalities to construction workers [1,2]. Despite the concerted effort made by various stakeholders, construction safety performance remains unsatisfactory. In 2019, the construction industry recorded 2947 accidents and 16 fatalities, accounting for 31.8% and 72.7% of overall industrial accidents and fatalities in Hong Kong, respectively [3]. Similar situations were observed in other regions, including the UK and the US [4]. Sousa et al. [5] stated that construction workers are subjected to three times the probability of dying and twice the probability of being injured compared with the average of workers in all other industrial sectors. The high personal, social, and financial costs resulting from construction injuries and fatalities has demanded safety researchers’ attention for improving construction safety performance.

Scholars have developed a variety of approaches for enhancing construction safety performance. Generally, these approaches can be classified into three domains, namely technology-based approaches, management-based approaches, and behavior-based approaches. For example, Fang et al. [6] proposed an innovative method of establishing an as-built virtual environment to facilitate safety training for crane operators. Guo et al. [7] developed a new warning system that incorporates building information modeling and positioning technology for monitoring unsafe on-site behavior of workers to boost construction safety management. Ahn et al. [8] developed an innovative method of delivering safety training to construction workers with the use of 3D simulation technology, which is effective in helping workers to actively learn safety knowledge and increase their enjoyment during the training. Salmi et al. [9] designed sensor-based robots for building construction to reduce the exposure of construction workers to risky work conditions. Yeo et al. [10] proposed a new measure to quantitatively evaluate the effectiveness of Internet of Things technologies for construction-accident prevention. In Hong Kong, the construction industry has been dedicated to developing innovative technology to improve construction safety performance, such as virtual reality technology for safety training [11] and modular integrated construction technology for enhancing production control and safety [12].

In addition to technology-based approaches, researchers have focused on management-based approaches for improving construction safety. For instance, Ismail et al. [13] identified factors that influence implementing a construction safety management system and reported that the most influential safety factors were personal awareness and communication. Jazayeri and Dadi [14] provided an overview of various construction safety management systems, such as safety promotion, management plans, risk management, and hazard identification. Tang et al. [15] developed a management system for providing personalized and real-time safety instructions to construction workers. Alruqi and Hallowell [16] conducted a meta-analysis study to investigate the relationship between leading safety indicators (which are used to assess the safety management system of an organization) and the safety performance of construction workers. Trinh et al. [17] proposed an innovation framework to measure the resilient safety culture of the construction industry, which can help improve the safety performance of construction workers. Pereira et al. [18] examined the relationships between safety-management system factors and accident precursors and found that accident precursors can result from project reworks, schedule pressure, and change orders. Choe et al. [19] examined the discrepancy in construction safety management practices and gave recommendations for developing effective safety management practices to enhance construction safety.

As for behavior-based approaches, researchers attempted to understand the behavior of construction workers. For instance, Seo et al. [20] proposed a behavioral model to explain construction worker safety behavior by using structural equation modeling (SEM). Guo et al. [21] developed a research model to predict construction worker safety behavior. Man et al. [22] qualitatively explored the experiences and attitudes of construction workers toward risk-taking behaviors and identified the crucial factors that accounted for their risk-taking behavior. On the basis of previous findings (Man, Chan and Wong [22], Man et al. [23] subsequently examined how personal (outcome expectancy, perceived behavioral control, attitude toward risk-taking behavior, and risk perception) and organizational (safety promotion policy, safety training, and work stress) factors influence the risk-taking behavior of construction workers.

The occurrence of industrial accidents results from a combination of different factors, such as organizational factors [24], psychological factors [25], and unsafe behavior [26]. As a result, understanding the unsafe behavior of workers is useful to discourage risky behavior, thus reducing the occurrence of industrial accidents. Risk-taking behavior, one of unsafe behavior, refers to engaging in activities that involve potential harm or danger for a chance to gain certain benefits [27,28]. Man, Chan and Wong [22] and Low et al. [29] emphasized that understanding construction worker risk-taking behavior is important for developing effective safety interventions to decrease the occurrence of construction accidents. Furthermore, Hasanzadeh et al. [30] used a mixed-reality roofing simulation to explore the relationship between safety interventions and risk-taking behaviors of roofing workers with the mediation of risk propensity and found that mediation was significantly supported. The safety climate has long been considered an essential organizational factor for explaining worker safety behavior [31]. In addition, the concept of the optimism bias has been used by traffic safety researchers to understand safety driving behavior [32]. Although some safety academics have exerted substantial efforts to gain insights into construction worker risk-taking behavior from the perspective of human factors [21,22], no studies have empirically examined how optimism bias and safety climate influence construction worker risk-taking behavior, leaving a research gap for the researchers of safety science. Therefore, this study aimed to address this research gap to contribute to the relevant literature. Given the findings of this study, construction safety practitioners can be expected to develop effective safety interventions for reducing the risk-taking behavior of construction workers, thereby decreasing the occurrence of accidents in the construction industry.

## 2. Research Model and Hypotheses

### 2.1. Optimism Bias

Different definitions of optimism bias are available in the literature. For instance, in the context of psychology, Weinstein [33] notes that that optimism bias is the tendency of individuals to believe that they are more skilled than their peers. Similarly, Klein and Helweg-Larsen [34] referred to an optimism bias as the tendency of people to think that their risk is lower than that of their peers. Sharot [35] defined optimism bias as the discrepancy between people’s expectations and the outcome that follows. In this study, two types of optimism bias of construction workers were considered, including one related to work risks and another related to hazard perception skills. Optimism bias related to work risks refers to the tendency of construction workers to think their work risks are lower than those of their peers. Optimism bias as related to hazard perception skills describes the tendency of construction workers to believe that they are more skilled at perceiving hazards at work than their peers.

The concept of optimism bias is applicable to various research fields, such as technology acceptance [36], transportation safety [37], construction safety [38], and project management [39]. In the transportation safety research area, White et al. [37] discovered that young drivers who believed that they are less likely to have an accident and who are more skilled at driving than their peers perform less precautionary driving behavior and more dangerous driving behavior. In the construction safety literature, the optimism bias has been recognized as important in construction safety because of its association with construction worker precautionary behavior [38]. However, no studies examined the effect of optimism bias related to work risks and optimism bias related to hazard perception skills on construction worker risk-taking behavior. Therefore, according to the abovementioned theoretical knowledge obtained from previous studies, the following hypotheses about optimism bias related to work risks and optimism bias related to hazard perception skills were developed.

**Hypotheses** **1** **(H1).** *Optimism bias related to work risks positively influences risk-taking behavior of construction workers.*

**Hypotheses** **2** **(H2).** *Optimism bias related to hazard perception skills positively influences risk-taking behavior of construction workers.*

### 2.2. Safety Climate

Zohar [40] first proposed safety climate for understanding the occupational behavior of workers in industrial organizations. A safety climate is often regarded as the shared perceptions of workers about their organization’s safety practices, policies, and procedures [41]. Two major strategies are used to quantify a safety climate. One aims to develop organization- and industry-specific measurements of safety climate, which can reflect the characteristics of the organizational and/or industrial context (e.g., [42], whereas the other prefers the development of general or universal measurements of safety climate [43]. The latter provides the chance to understand safety climate’s antecedents and consequences in different languages, cultures, and contexts [44].

In the literature on occupational safety, a safety climate is identified as aa significant predictor linked to the safety performance of workers [45]. In construction safety research, researchers have extensively investigated the relationship between safety outcomes of construction workers and safety climate. For instance, safety climate negatively affected injuries and near misses among US construction workers [46], Hong Kong construction workers [47], Ontario construction workers [48], and construction workers in mainland China [49]. Apart from the safety outcomes of workers, a safety climate has been widely found to positively influence construction worker safety behaviors [43,49,50]. Yule et al. [50] examined the role of a safety climate in reducing power station workers’ risk-taking behavior and found that with good safety climate, power station workers perform less risk-taking behavior. However, how safety climate influences construction worker risk-taking behavior has not been examined in the relevant literature. Accordingly, the following hypothesis about safety climate was developed.

**Hypotheses** **3** **(H3).** *Safety climate negatively influences the risk-taking behavior of construction workers.*

### 2.3. Research Model

**Figure 1:**
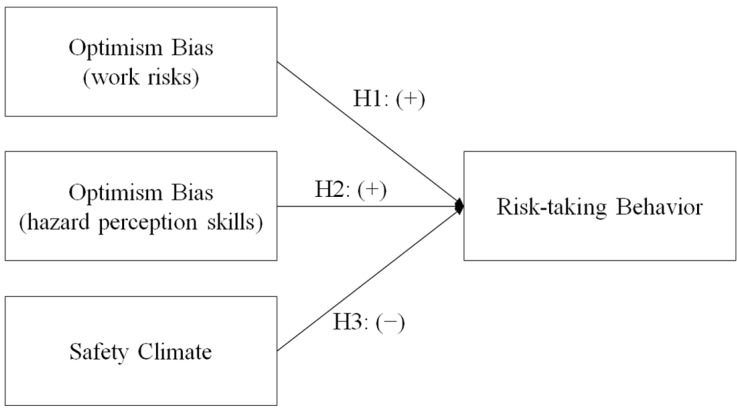
Research model to be tested in the current study.

In this study, the abovementioned literature review was used to develop the hypotheses for investigating the influence of optimism bias and safety climate on construction worker risk-taking behavior. Figure 1 presents the research model with the developed hypotheses.

## 3. Methodology

### 3.1. Research Design

A survey with a structured questionnaire was conducted to collect data. The effect of the optimism bias and of a safety climate on construction worker risk-taking behavior was examined by testing the hypotheses formulated in Section 2 using the collected data. The details about questionnaire development, participants, and data analysis are given below.

### 3.2. Questionnaire Development

A questionnaire survey is a method of collecting empirical data and can contain measurement items which are used to infer latent variables, for example, the optimism bias. The questionnaire consisted of four sections. The first section included 10 items about optimism bias related to work risks and four items about the optimism bias related to hazard perception skills, which were adapted from the transportation safety studies of Gosselin et al. [51] and White, Cunningham and Titchener [37], respectively, to fit the context of the current study. A 7-point Likert-type scale format ranging from 1 = “very unlikely” to 7 = “very likely” was used to measure optimism bias related to work risks, whereas a 7-point Likert-type scale format ranging from 1 = “much less” to 7 = “much more” was used to measure optimism bias related to hazard perception skills. The item score of optimism bias related to work risks was recoded inversely for an easy and intuitive interpretation of the results. As a result, high total scores on optimism bias related to work risks and optimism bias related to hazard perception skills indicate that respondents have high levels of optimism bias related to work risks and optimism bias related to hazard perception skills. The second section contained eight items measuring safety climate, adopted from the study of Huang, Lee, Chen, Perry, Cheung and Wang [43]. A high total score on the safety climate scale reflects that the organization in which the respondent works has a good safety climate. The third section had six items for measuring risk-taking behavior, adopted from the study of Rundmo [52]. The items related to risk-taking behavior were activities that involve potential harm or danger for a chance of gaining certain benefits, for example, “to get the job done quickly, you often ignore the safety rules.” A higher total score on the risk-taking behavior scale indicates that respondents often take risks at work. In the last section, a set of demographic questions, including age, gender, marital status, education level, and work experience in the construction industry, were presented. Before the questionnaires were distributed to construction workers, a pilot study was conducted by asking five safety experts who had more than 10 years of work experience in construction safety to provide comments on the item contents of the questionnaire. All the experts reported that the item contents were understandable and appropriate. Table 1 summarizes the item contents of optimism bias related to work risks, optimism bias related to hazard perception skills, safety climate, and risk-taking behavior.

### 3.3. Participants

A convenience-sampling technique was used to select construction workers in this study, and construction site visits were conducted to identify the workers. A total of 183 construction workers participated in this study. All 183 construction workers were from Hong Kong. As Hong Kong construction workers have a high job mobility for different construction companies due to the contracting or subcontracting nature of the industry, these 183 participants worked for different projects in the construction industry, and they are not the direct employees of any one company. The number of samples of this study (183) was considered suitable for the structural equation modeling because the mean sample size of construction research using structural equation modeling was 162 [53]. The designed questionnaire was distributed to participants during the site visits. To minimize the potential response bias, the participants were told before answering the questionnaire that they have the right to quit this research activity, and all the gathered information would be managed confidentially. Written informed consent was provided by the participants. As shown in Table 2, of the 183 participants, 177 were male (96.72%) and six were female (3.28%). They were aged from 23 to 62. Most of them had a lower secondary education level or above (78.69%) and had worked in the construction industry for at least one year (98.91%).

### 3.4. Data Analysis

The research model with the hypotheses formulated in Section 2 was tested using SEM. According to Kline [54], the advantages of using SEM include: (a) the relationships among variables can be estimated with the consideration of measurement errors; (b) SEM considers measurement model (i.e., confirmatory factor analysis [CFA] for assessing measurement properties) and structural model (for assessing how factors influence another), resulting in a robust analytic approach; (c) SEM can deal with latent variables which cannot be observed directly; and (d) SEM can simultaneously examine hypothesized models while considering the entire system of variables. Therefore, SEM has been widely used in construction safety studies [23,55]. A measurement model was used to examine the relationship between latent variables and their measurement items while a structural model was used to examine the relationship between the latent variables.

Prior to SEM, the psychometric properties of the scales were evaluated using CFA, including construct validity, convergent validity, discriminant validity, and internal consistency reliability. CFA is a useful statistical method for behavioral sciences because of its ability to provide information on whether the data fit the measurement model and to identify poor items of the measurement [56]. Construct validity is confirmed if model fitness indices achieved the required levels. Following the recommendations by Kline [54], four model fitness indices were included, namely, the ratio of Chi-square value to degree of freedom (*χ*^2^/*df*), Tucker–Lewis Index (TLI), comparative fit index (CFI), and root mean square error of approximation (RMSEA). The required levels included *χ*^2^/*df* < 5, CFI > 0.9, TLI > 0.9, and RMSEA < 0.08 [54,57]. The convergent validity and discriminant validity of the measurement were also examined. Convergent validity refers to the degree to which two measures of constructs that are theoretically related to one another are actually related [58]. The convergent validity of the measurement is acceptable if the factor loading (FL) of an item on its designed construct, composite reliability (CR) for each construct, and the average variance extracted (AVE) for each factor is greater than 0.7, 0.7, and 0.5, respectively. Discriminant validity is the extent to which the constructs are different empirically [58]. Discriminant validity is acceptable when each construct has the value of the square root of AVE greater than the correlations among the constructs of the research model [59]. Additionally, this study used Cronbach’s alpha to assess the internal consistency reliability of the measurement for each construct [60]. Internal consistency reliability is acceptable if the value of the Cronbach’s alpha is higher than 0.7.

After the confirmation of the reliability and validity of the measurement, SEM was used to test the hypotheses in the proposed model. The model fitness indices and required levels are the same as those in CFA (i.e., *χ*^2^/*df* < 5, CFI > 0.9, TLI > 0.9, and RMSEA < 0.08). The CFA and SEM were performed using AMOS 21 software (IBM, Armonk, YN, USA).

## 4. Results

### 4.1. Descriptive Statistics Related to Optimism Bias

The mean scores of the optimism bias related to hazard perception skills and optimism bias related to work risks were 4.11 (SD = 1.49) and 3.63 (SD = 1.73), respectively. The mean score of the optimism bias related to hazard perception skills was not significantly different (*p* = 0.300), whereas that of optimism bias related to work risks was significantly different (*p* = 0.004) from the score of 4.0, which represented neutrality (*i.e.*, midpoint) on the scale.

### 4.2. Measurement Model

The results of the measurement model fit assessment (Table 3) indicated that all model-fit index values achieved the recommended criteria. Specifically, the measurement model can adequately account for the collected data. Table 4 reveals that all constructs had Cronbach’s alpha values that ranged from 0.86 to 0.98. These values were larger than the critical requirement of 0.7, implying that the internal consistency reliability of all constructs is acceptable [61]. In addition, all items had FL values greater than 0.7. All constructs had CR values that exceeded 0.7. The AVE value of each construct was between 0.60 and 0.84, exceeding 0.5. Thus, the convergent validity of the measurement was acceptable. Table 5 shows that all constructs had a square root of the AVE greater than the correlations among constructs. Therefore, the acceptable discriminant validity of the measurement was confirmed. In conclusion, the measurement model assessment demonstrated an adequate model fit, high internal consistency reliability, and acceptable convergent and discriminant validity, reflecting that SEM was appropriate for testing the hypotheses in the research model.

### 4.3. Structural Model

**Figure 2:**
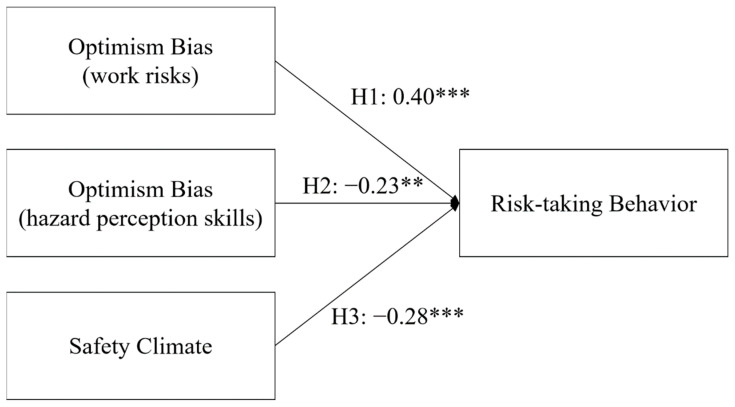
Proposed research model with hypothesis testing results (the values are standardized path coefficients; **: *p* < 0.01; and ***: *p* < 0.001).

SEM was used to test the proposed research model (Fig. 1) and to infer the hypotheses of interest. Table 3 shows that *χ*^2^/*df*, CFI, TLI, and RMSEA achieved the criteria. Thus, the hypothesized relationships can be sufficiently represented by the research model. In testing for the developed hypotheses, the results indicated that two hypotheses were supported (Table 6). Specifically, the optimism bias related to work risks has a positive influence (H1), whereas a safety climate has a negative influence on risk-taking behavior (H3). Although H2, which states that optimism bias related to hazard perception skills positively influences risk-taking behavior was significant, the standardized path coefficient of H2 was negative. Therefore, this hypothesis was not supported. Figure 2 shows the proposed model with the results (Table 6).

## 5. Discussion

This study empirically investigated the influence of optimism bias and safety climate on construction worker risk-taking behavior. Theoretical contributions and practical implications can be provided by this study, which are discussed below, followed by limitations and future research.

### 5.1. Theoretical Contributions

First, this study found that, on average, construction workers perceive that they have a similar level of hazard perception skills as their peers, but that they have a higher likelihood of encountering an accident than their peers. Moreover, the optimism bias related to work risks positively influences construction worker risk-taking behavior, similar to the previous finding that the optimism bias related to accident risk leads drivers to take less precautionary behavior and engage in more unsafe driving behavior [37]. Construction workers who hold a high level of optimism bias related to work risks tend to take risks when they work at construction sites. This finding complies with that of Man, Chan and Wong [22] who reported that construction workers who perceive a low level of risks tend to take risks at work. A previous study by Caponecchia and Sheils [38] found that optimism bias related to work risks did not correlate to the safe work behavior of Australian construction workers. After the previous work of Caponecchia and Sheils [38], no follow-up study was conducted to investigate the influence of optimism bias related to work risks on the risk-taking behavior of construction workers. The current work successfully addressed this research gap, enriching the relevant literature on construction safety. Besides, the current study provided evidence in the context of construction safety for the statement made by Weinstein [64], that the optimism bias related to accident risk may remarkably reduce the attempts of the public to perform risk-reducing behavior in the context of future life events. However, less knowledge about how to effectively reduce optimism bias related to work risks of construction workers is available in the literature. This research area should be paid further attention from construction safety researchers.

Second, the optimism bias related to hazard perception skills negatively affects risk-taking behavior, contrary to the finding of White, Cunningham and Titchener [37] who noted that drivers who consider themselves more skillful in driving are more likely to drive riskily. One possible reason for this phenomenon is that construction workers with a high tendency to believe that they are more skilled in perceiving hazards at work than their peers may also have a high level of risk perception. In the correlation analysis (Table 4), optimism bias related to hazard perception skills was significantly negatively associated with optimism bias related to work risks. Thus, construction workers who believed they are more skilled in hazard perception than their peers perceive a greater level of risks at work and are more prudent and cautious at work than their peers. This result indirectly supported the tentative explanation for the surprising phenomenon. Man et al. [65] recently developed and validated a measurement for quantifying the risk perception of construction workers. The measurement can be used to investigate how the relationship between optimism bias and construction worker risk-taking behavior was mediated by risk perception in the future to contribute to the relevant literature. Therefore, more research effects should be made to obtain substantial theoretical evidence to support the tentative explanation and complement the results of the current study.

Third, a safety climate has long been a crucial factor in construction safety [66,67,68]. However, studies on how it influences construction worker risk-taking behavior are lacking. This study successfully addressed this research gap. The results revealed that safety climate negatively influences construction worker risk-taking behavior. Specifically, a good safety climate can lead construction workers to perform less risk-taking behavior. This study served as the first attempt to examine this research area and confirmed that safety climate is a crucial factor in discouraging construction workers from engaging in risk-taking behavior. Moreover, the underlying mechanism of how safety climate influences construction worker risk-taking behavior is interesting to examine. Specifically, the mediators in the relationship between safety climate and construction worker risk-taking behavior should be explored in the future.

### 5.2. Practical Implications

The current study demonstrated that optimism bias and safety climate significantly influence construction worker risk-taking behavior. According to the findings, practical suggestions were made to reduce construction worker risk-taking behavior. First, construction management must be aware that the optimism bias, related to work risks, positively affects construction worker risk-taking behavior. First-aid training should be provided to construction workers who have a high level of optimism bias related to work risks, because Lingard [69] affirmed that first aid training can lower construction workers’ mindset that “it will not happen to me.” Construction workers who receive first aid training are expected to perceive that they are more likely to encounter work-related illness or injury and express considerable safety concerns, thereby reducing their risk-taking behavior. Therefore, first aid training or at least a few hours of training using an automated external defibrillator is recommended for the mandatory basic safety training. Those who complete training should receive certification (commonly known as “Green Card” in Hong Kong), which makes people eligible to be employed by construction companies. Second, the importance of safety climate in preventing construction workers from working unsafely should be continuously emphasized in the construction industry. Concerned authorities and stakeholders can organize activities such as safety meetings and safety award presentations [70] to cultivate a good safety climate for the industry. Besides, training and the preventive action of supervisors positively influence safety climate [71]. Third, a safety-offence points system is a useful method to change workers’ behavior [72,73] and should be developed in the construction industry to reduce construction workers’ risk-taking behavior. In the safety-offence points system, construction workers who perform risk-taking behavior at work can then receive safety–offence points. They will be subject to attending safety training and monetary penalty when their safety–offence points are high. Thus, practitioners can develop innovative interventions that encourage such actions of supervisors to improve safety climate. When construction workers perceive good safety climate, their intention to task risks at work can be reduced.

### 5.3. Limitations and Future Research

Although this study obtained significant findings, its limitations should be recognized. First, the current study used a cross-sectional survey to collect the data. Future research can collect longitudinal data to gain an in-depth understanding of how safety climate and optimism bias affect the risk-taking behavior of construction workers over time. Second, the current study only considered the general safety climate. Li et al. [74] suggested that the safety climate for the construction industry has six dimensions, including co-workers’ interaction, workers’ self-perception of safety, safety environment, workers’ involvement in safety, safety personnel support, and safety management involvement. These dimensions of safety climate were not considered in this study. The way these dimensions affect construction worker risk-taking behavior should be examined in the future. Third, the participants of this study were from Hong Kong. Selecting samples of construction workers from different regions and countries is important to make the research conclusions more general. Therefore, future studies can recruit construction workers from different regions and countries to compare the optimism bias of construction workers. Last, there were only seven female construction workers involved in this study. The gender effect on the relationship between optimism bias and risk-taking behavior of construction workers is an interesting research topic and should be investigated in future studies.

## 6. Conclusions

The risk-taking behavior of construction workers has received increasing attention from construction safety researchers. Given that worker risk-taking behavior reduces construction safety performance, this study successfully obtained theoretical and practical implications about construction worker risk-taking behavior from the perspective of optimism bias and safety climate. The optimism bias, related to work risks, positively influences construction worker risk-taking behavior, whereas safety climate and optimism bias related to hazard perception skills negatively affect risk-taking behavior. According to the results, construction practitioners and the concerned authorities can develop effective safety interventions and policies for reducing construction worker risk-taking behavior, thereby decreasing the occurrence of construction accidents.

## Figures and Tables

**Table 1:** Item contents of the constructs.

Constructs	Items	Contents
Optimism bias related to work risks (OBWR)		If you experience the following situations, how likely do you think will you encounter an accident compared with other construction workers of the same age and gender as you?
	OBWR1	Lifting or carrying items
	OBWR2	Working without safety shoes
	OBWR3	Working at heights without safety belts
	OBWR4	Working without a helmet
	OBWR5	Using mobile phones while working
	OBWR6	Working with electricity but without insulated gloves
	OBWR7	Working under the lifting route
	OBWR8	Touching an operating machine or the object in the machine
	OBWR 9	Handling sharps without cut-proof gloves
	OBWR 10	Working on the road at night without a reflective vest
Optimism bias related to hazard perception skills (OBHPS)		Compared with other construction workers of the same age and gender as you, how skillful are you at…
	OBHPS1	Promptly detecting dangerous things?
	OBHPS2	Perceiving dangerous things with sufficient time to react?
	OBHPS3	Noticing various dangerous things at the same time?
	OBHPS4	Responding to multiple potentially dangerous things at the same time?
Safety climate (SC)	SC1	Your senior manager tries to improve the safety level of each department continuously.
	SC2	Your senior manager requests each manager to improve the safety of their department.
	SC3	Your senior manager uses any available information to improve the existing security rules.
	SC4	Your senior manager provides employees with a lot of safety information.
	SC5	Your supervisor discusses how to improve the safety level of the site with your co-workers.
	SC6	Your supervisor lets employees work safely by explanation instead of by command.
	SC7	Your supervisor reminds employees to work safely.
	SC8	Your supervisor ensures you comply with all safety rules (not only the important ones).
Risk-taking behavior (RTB)		In your daily work, to get the job done
	RTB1	quickly, you often ignore the safety rules.
	RTB2	You often do some illegal behaviors.
	RTB3	You often do the work improperly.
	RTB4	You often take risks to complete your work.
	RTB5	You often do not use personal protective equipment.
	RTB6	To get the job done quickly, you often do not follow the right job.

**Table 2:** Participant demographic information (*n* = 183).

Variables	Categories	Frequencies	Percentage (%)
Gender	Male	177	96.72
	Female	6	3.28
Age	20–29	16	8.74
	30–39	41	22.40
	40–49	47	25.68
	50–59	36	19.67
	>59	8	4.37
	Unspecified	35	19.14
Marital status	Single	65	35.52
	Married	89	48.63
	Divorced	6	3.28
	Widowed	2	1.09
	Unspecified	21	11.48
Education level	Primary school	22	12.02
	Lower secondary	41	22.40
	Higher secondary	96	52.46
	Post-secondary	7	3.83
	Unspecified	17	9.29
Work experience (years) in the construction industry	<1	2	1.09
	1–5	78	42.62
	6–10	49	26.78
	11–15	30	16.39
	16–20	8	4.37
	21–30	1	0.55
	Unspecified	15	8.20

“Unspecified” means no responses provided by participants.

**Table 3:** Results of the measurement model and structural model assessments.

Model Fit Indices	Measurement Model	Structural Model	Recommended Values	Results	References
*χ*^2^/*df*	1.32	1.32	< 5	Acceptable	Hair et al. [62] Kline [54] McDonald and Ho [63]
CFI	0.98	0.98	> 0.9	Acceptable	
TLI	0.98	0.98	> 0.9	Acceptable	
RMSEA	0.04	0.04	< 0.08	Acceptable	

**Table 4:** Results of the convergent validity and reliability assessment.

Constructs	Items	Mean	SD	FL	AVE	CR	Cronbach’s Alpha
Optimism bias related to work risks (OBWR)	OBWR1	3.91	2.08	0.70	0.68	0.95	0.95
	OBWR2	3.74	1.97	0.85			
	OBWR3	3.47	2.07	0.84			
	OBWR4	3.59	2.14	0.86			
	OBWR5	3.56	1.96	0.83			
	OBWR6	3.45	2.14	0.88			
	OBWR7	3.51	2.17	0.84			
	OBWR8	3.82	1.98	0.81			
	OBWR9	3.51	1.93	0.84			
	OBWR10	3.72	2.11	0.75			
Optimism bias related to hazard perception skills (OBHPS)	OBHPS1	4.07	1.86	0.76	0.60	0.86	0.86
	OBHPS2	4.12	1.81	0.79			
	OBHPS3	4.16	1.68	0.78			
	OBHPS4	4.10	1.79	0.77			
Safety climate (SC)	SC1	5.47	1.67	0.92	0.84	0.98	0.92
	SC2	5.48	1.70	0.92			
	SC3	5.46	1.70	0.92			
	SC4	5.54	1.67	0.91			
	SC5	5.56	1.61	0.92			
	SC6	5.45	1.74	0.91			
	SC7	5.57	1.73	0.92			
	SC8	5.54	1.81	0.92			
Risk-taking behavior (RTB)	RTB1	2.63	1.50	0.70	0.67	0.92	0.92
	RTB2	2.44	1.49	0.88			
	RTB3	2.44	1.41	0.84			
	RTB4	2.26	1.33	0.84			
	RTB5	2.34	1.45	0.85			
	RTB6	2.37	1.33	0.80			

**Table 5:** Results of the discriminant validity assessment.

	OBWR	OBHPS	SC	RTB
OBWR	**−0.82**			
OBHPS	−0.17 *	**−0.77**		
SC	−0.05	−0.38 ***	**−0.92**	
RTB	−0.46 ***	−0.19 *	−0.22 **	**0.82**

The values in bold type are square roots of AVE values for corresponding constructs; the other values are correlations among constructs; OBWR means optimism bias related to work risks; OBHPS means optimism bias related to hazard perception skills; SC means safety climate; RTB means risk-taking behavior; *: *p* < 0.05; **: *p* < 0.01; and ***: *p* < 0.001.

**Table 6:** Hypothesis testing results.

Hypotheses	Standardized Path Coefficients	*p*-Values	Results
H1: Optimism bias related to work risks positively influences risk-taking behavior.	0.40	<0.001	Supported
H2: Optimism bias related to hazard perception skills positively influences risk-taking behavior.	−0.23	<0.01	Not supported
H3: Safety climate negatively influences risk-taking behavior.	−0.28	<0.001	Supported

## Data Availability

The data presented in this study are available on request from the corresponding author.
